# Smooth Muscle Cell Specific Activity of SGK-1 Alters Aortic Stiffness and Abdominal Aortic Aneurysm Growth

**DOI:** 10.3390/jvd5010010

**Published:** 2026-02-16

**Authors:** Matthew Anderson, Leilei Zhang, Mario Figueroa, Victoria Mattia, Alexander Rovner, Vinitha Uppalapati, Ying Xiong, Rupak Mukherjee, Jeffrey A. Jones, Jean Marie Ruddy

**Affiliations:** 1 Division of Vascular Surgery, Medical University of South Carolina, Charleston, SC 29425, USA; 2 Division of Cardiothoracic Surgery, Medical University of South Carolina, Charleston, SC 29425, USA; 3 RalphHJ Johnson Veterans Affairs Health Care System, Charleston, SC 29401, USA

**Keywords:** aneurysm, biomarkers, calcification, aortic stiffness

## Abstract

**Background/Objective:**

Activity of SGK-1 has been associated with mechanical aspects of vascular remodeling and matrix stiffening has been a known characteristic of AAA. We hypothesis that VSMC-specific SGK-1 activity is vital to growth of AAA and contributes to progressive aortic stiffness.

**Methods:**

C57Bl/6 and SMC-SGK-1KO^+/−^ mice underwent AAA induction vs Sham on day 0. A subset of C57Bl/6 mice had pump implantation to treat with EMD638683. Aortic ultrasound images were obtained on Day 0 and Day 21 and analyzed for mechanical parameters. At terminal procedure the infrarenal aorta was harvested for immunoblot analysis.

**Results:**

At Day 21, C57Bl/6+AAA mice showed growth of 72.27% ± 2.2% versus the C57Bl/6+Sham (*p* < 0.0001) with associated 3.71 ± 1.15-fold increase in SGK-1 activity (*p* = 0.001). C57Bl/6+AAA+EMD mice demonstrated growth of 23.68% ± 2.82% (*p* = 0.0452) with no significant change in SGK-1 activity. SMC-SGK1-KO^+/−^+AAA mice had growth of 28.20% ± 3.74% compared to SMC-SGK1-KO^+/−^+Sham (*p* = 0.004) with increased SGK-1 activity (*p* = 0.0303). Radial strain was significantly reduced in the C57Bl/6+AAA (*p* = 0.0062) and C57Bl/6+AAA+EMD (*p* = 0.0135) when compared to C57Bl/6+Sham. Distensibility was significantly reduced in C57Bl/6+AAA (*p* = 0.01). Pulse propagation velocity (PPV) was significantly elevated in C57Bl/6+AAA mice (*p* < 0.0001) but inhibited by EMD therapy (*p* = 0.0007 vs. C57Bl/6+AAA). SMC-SGK1-KO^+/−^ +AAA mice showed significant reductions in radial strain (*p* = 0.0011) and distensibility (*p* = 0.0233) with a modest, but significant, increase in PPV (*p* = 0.0148).

**Conclusions:**

SGK-1 inhibition attenuated AAA growth and preserved vascular function. Targeting this pathway may provide a directed medical therapy for AAA and warrants further investigation.

## 1. Introduction

Abnormal dilation of the aorta, known as abdominal aortic aneurysms (AAA) is known to affect up to 8% of the population in the U.S., with the incidence of AAA doubling for every decade of life greater than age 65 [1,2]. Ruptured aneurysms have mortality up to 80%, even when patients are able to present to the hospital for emergent repair. Given most AAAs are asymptomatic until rupture, early detection via screening is an important strategy for prevention of rupture [3]. National imperatives have led to widespread screening programs and progressive use of stent graft therapy, but opportunities for meaningful medical management of AAA have remained elusive. A challenge to targeting AAA growth is rooted in the complex interplay of genetic, environmental, and hemodynamic contributors which drive the progressive atherosclerotic inflammation, matrix remodeling, and vascular smooth muscle cell (VSMC) apoptosis known to occur [4–6]. Considering the intrinsic hemodynamic forces to be a common denominator, identification of key mechanotransduction pathways may facilitate development of druggable targets to reduce AAA growth.

Stretch-induced remodeling in other vascular pathologies, such as vein graft intimal hyperplasia and pulmonary HTN, has been extensively studied and accredited to the activity of the serum and glucocorticoid inducible kinase-1 (SGK-1) [7,8], a kinase linked to modulation of proteases and pro-inflammatory cytokines [9,10]. Specifically within the abdominal aorta, a murine hypertension model has demonstrated that VSMC activity of SGK-1 was instrumental in the production of Interleukin-6 and Monocyte Chemoattractant Protein-1, thereby driving macrophage accumulation [11]. This inflammatory infiltrate is expected to contribute to the degenerative remodeling and subsequent changes in aortic stiffness [12]. Recognizing the epidemiologic association with hypertension representing a major risk factor for AAA development, extending the investigation of SGK-1 to account for mechanical aspects of aneurysmal degeneration is warranted. Interestingly, utilizing RNA sequencing in a rabbit aneurysm model, expression of this mechanosensitive kinase was upregulated greater than two-fold [13], further indicating that SGK-1 may contribute to degenerative remodeling. Herein we propose to address the hypothesis that VSMC-specific SGK-1 activity is vital to growth of AAA and contributes to progressive aortic stiffness.

## 2. Materials and Methods

### 2.1. Animal Care and Use

All animal care and surgical procedures were approved by the Ralph H. Johnson Department of Veterans Affairs Health Care System Institutional Animal Care and Use Committee (ACORP#6414) as well as the Medical University of South Carolina Institutional Animal Care and Use Committee (AR#2020–01052). Wild-type mice of the C57Bl/6 were purchased from Jackson Laboratories (Bar Harbor, ME, USA). Experimentation was supported by a local breeding colony. Additionally, MyH11^Cre-EGFP^ mice were purchased from Jackson Laboratories (Bar Harbor, ME, USA) and crossed with SGK-1^flox/flox^ mice from the Fejes-Toth laboratory (Dartmouth College, Hanover, NH, USA) [14] lab to create a heterozygous smooth muscle-cellspecific SGK-1 knockout strain (SMC-SGK-1KO^+/−^). Each transgenic strain had been established on a C57Bl/6 background and local breeding colonies were established. Confirmation of genotyping for the SMC-SGK-1KO^+/−^ mice was completed through Transnetyx Inc. (Cordova, TN, USA). SMC-SGK-1KO^−/−^ mice were not bred for experimentation given previous reports of severe physiologic impairment and potential lethality [15,16]. Mice were randomly assigned to treatment groups in equal proportion by sex and age.

### 2.2. AAA Induction

Considering the rarity of open surgical AAA repair and this investigation’s interest in understanding early aspects of AAA development, a murine model was chosen rather than human tissue analysis, but pilot data did indicate elevated SGK-1 activity in human AAA. Anesthesia was delivered via 2% isoflurane and subcutaneous injection of 0.05 mg/kg buprenorphine. Intraoperatively, heart rate was continuously monitored. Mice (aged 12–20 weeks) then underwent midline laparotomy and the small bowel was mobilized to the right upper quadrant. The abdominal aorta was exposed from the left renal vein to the aortic bifurcation. Baseline aortic diameter (AoD) was measured using calibrated digital microscopy. With a sterile sponge, 0.5 M calcium chloride (CaCl_2_) solution was placed directly against the aortic adventitia for 10 min. The sponge was then removed and the abdomen was closed. Mice recovered in a heated and oxygenated chamber before being returned to their home cage. A random selection of the mice also underwent osmotic pump implantation as described below. NaCl (0.9%) was utilized for the sham procedure in C57Bl/6 and SMC-SGK-1KO^+/−^ mice.

### 2.3. Osmotic Mini-Pump Implantation

EMD638683 (MedChemExpress, Monmouth Junction, NJ, USA) is a selective inhibitor of SGK-1 with no identified alternate targets [17]. This chemical was dissolved in dimethyl-sulfoxide and diluted to achieve 2.5 mg/kg/day delivery via minipump. With random selection on post-op day one from aortic procedure, a subset of mice were anesthetized and underwent subcutaneous implantation of loaded Alzet osmotic minipump via a left flank incision (model 1004; Durect Corportation, Cupertino, CA, USA). EMD638683 was delivered for 21 days.

### 2.4. Ultrasound Image Acquisition and Analysis

Ultrasound images were obtained on Day 0 and Day 21 of treatment using a VisualSonics Vevo 3100 ultrasound machine (FUJIFILM VisualSonics, Toronto, ON, Canada). Using isoflurane to maintain a heart rate of 450–550 bpm, mice were anesthetized and placed supine on a warmed (42 °C) monitoring board. With a 55 MHz linear probe, B-mode ultrasound image loops of the abdominal aorta were optimized to one cardiac cycle. These images were captured using axial and longitudinal orientation through the center of the aorta. Imaging extent included renal vein to aortic bifurcation.

In the Vevo VascLAB software (V1) (FUJIFILM VisualSonics, Toronto, ON, Canada), vessel walls were labeled and tracked across one cardiac cycle. This enabled calculations of radial strain, distensibility, and pulse propagation velocity (PPV). Radial strain represented percent change in diameter between systole and diastole [12]. Using pulse pressure obtained during concurrent tail-cuff quantifications [12], distensibility was likewise calculated. PPV has been defined as the speed at which a pulse travels down the aortic wall, therefore it was quantified by the time between pulse images of the proximal and distal sections of the abdominal aorta. Notably, decreases in radial strain and distensibility correlate to increased aortic stiffness. Alternatively, increased PPV indicates aortic stiffness [18].

### 2.5. Aortic Tissue Harvest

Terminal procedures were conducted on Day 21. Anesthesia was induced with 2% isoflurane and midline laparotomy was performed. After mobilizing the small bowel, the aorta was exposed from the left renal vein to the aortic bifurcation. Aortic diameter measurements were taken using calibrated digital microscopy. This aortic segment was then resected and placed in sterile saline. The aorta was snap-frozen in a liquid nitrogen slurry. Frozen aortic samples were stored in −80 °C until further analysis.

### 2.6. Immunoblot Analysis

Relative abundance of SGK-1, phosphorylated SGK-1 (pSGK-1), and α-tubulin were determined by immunoblotting as previously described [19,20]. Briefly, aortas were homogenized and 20 μg of protein was fractionated on a 10% polyacrylamide gel by electrophoresis. The proteins were transferred to nitrocellulose membranes (0.45 μm; Bio-Rad, Hercules, CA, USA) and incubated in antisera specific for SGK-1 (ab32374 AbCam, Waltham, MA, USA; 1:1000), pSGK-1 (ab55281, AbCam, Waltham, MA, USA; 1:1000) and α-tubulin (ab7291, AbCam, Waltham, MA, USA; 1:1000) in 0.1% Tris-buffered saline with Tween. The secondary peroxidase-conjugated antibody was applied (1:5000; 0.1% Tris-buffered saline with Tween). Signals were detected with a chemiluminescent substrate (Western Lighting Chemiluminescence Reagent Plus; PerkinElmer, San Jose, CA, USA) and recorded on film. Band intensity was quantified using ImageJ 53C software (National Institute of Health, Bethesda, MD, USA).

### 2.7. Statistical Analysis

Abdominal AoD images acquired using calibrated digital microscopy Pax-IT software (V2) (Villa Park, IL, USA) were measured from outer wall to outer wall. Results represent the total AoD. Similar to previous work focused on tissue kinase activity [11,19,20], SGK-1 activity in the murine aorta was derived as the ratio of the OD-derived abundance of pSGK-1:totalSGK-1. This ratio was calculated for each aortic sample and the fold change established relative to the C57Bl/6 +Sham mice, which represent normal aortic physiology. Ultrasound-derived mechanical parameters were analyzed at each time point and data is represented as mean +/− standard error of the mean (SEM).

Data sets were analyzed by paired Student’s *t*-test or by ANOVA with post hoc mean separation using the Tukey correction for multiple comparisons. Statistical tests were performed using GraphPad Prism (GraphPad Software V10.6.1; Boston, MA, USA) with significance established at *p* < 0.05.

## 3. Results

### 3.1. SGK-1 Activity in AAA

**Figure 1.**
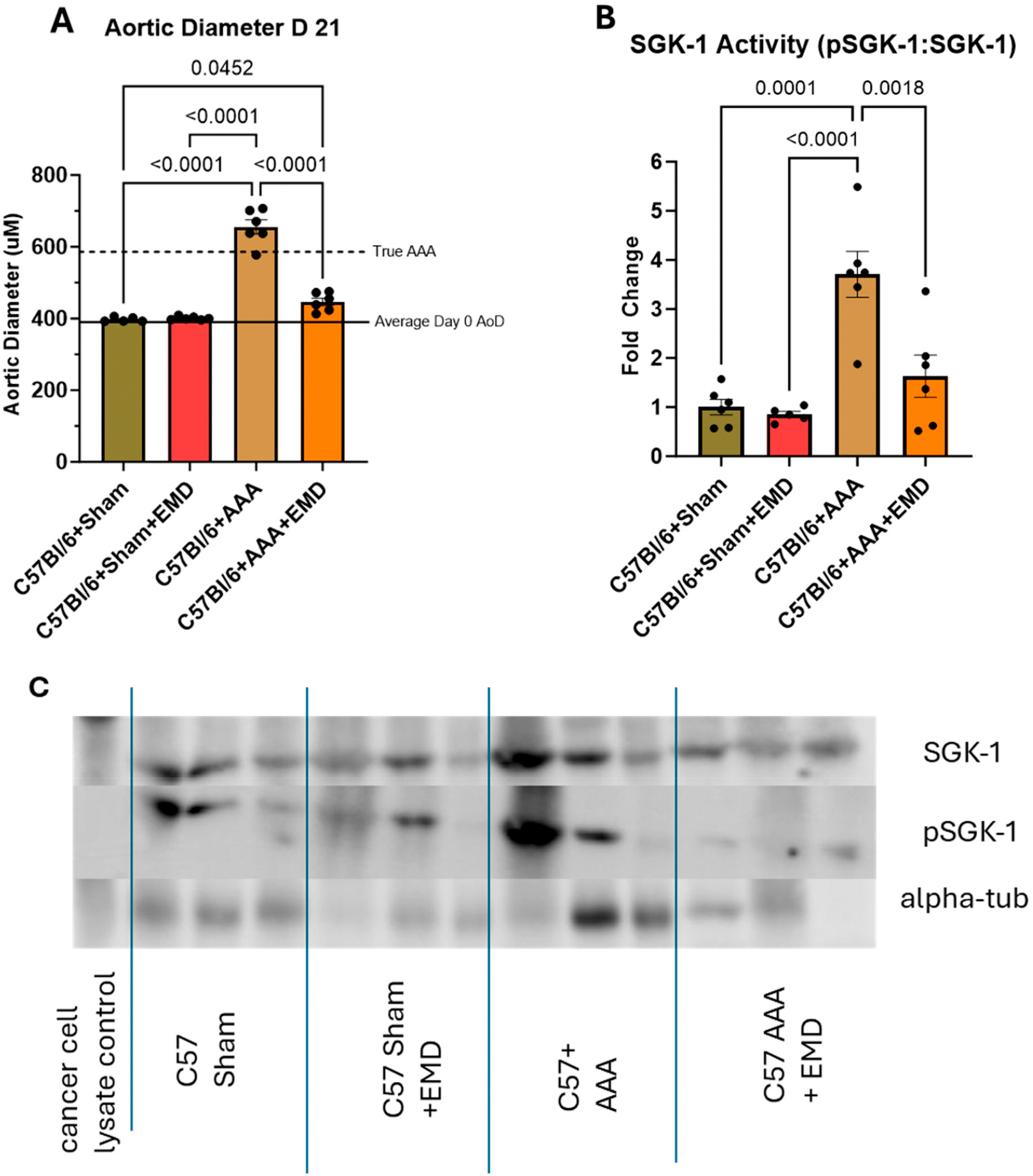
Panel (**A**): Aortic diameter (AoD) of C57Bl6 mice +/− AAA induction and +/− EMD treatment at day 21. Solid black line represents average baseline aortic diameter on day 0. Dotted black line represents an average 50% increase in diameter to represent the threshold of a true AAA. Panel (**B**): SGK-1 activity represented as fold change from C57Bl/6+Sham. Panel (**C**): Representative Western blot indicating abundance of pSGK-1 versus SGK-1 and alpha-tubulin. Statistical analysis represents ANOVA.

In the sham procedures, no change in AoD was detected in either the C57Bl/6+Sham or C57Bl/6+Sham+EMD groups. For the C57Bl/6+AAA cohort, average AoD increased to 655.17 μM ± 19.69 μM (Figure 1A), which represented an average diameter growth of 72.27% ± 2.2% compared to C57Bl/6+Sham (*p* < 0.0001). This aortic growth to small AAA was associated with a 3.71 ± 1.15-fold increase in SGK-1 activity as compared to the C57Bl/6+Sham (Figure 1B,C; *p* = 0.001). Accordingly, the C57Bl/6+AAA+EMD mice only demonstrated an average AoD increase to 446.16 μM ± 10.41 μM, representing an average diameter growth of 23.68% ± 2.82% compared to the C57Bl/6+Sham (*p* = 0.0452), and coinciding with only 1.63 ± 1.05-fold increase in SGK-1 activity which was not statistically significant as compared to the C57Bl/6+Sham (*p* = NS) and suggests that SGK-1 activity promoted progressive aortic dilation.

**Figure 2.**
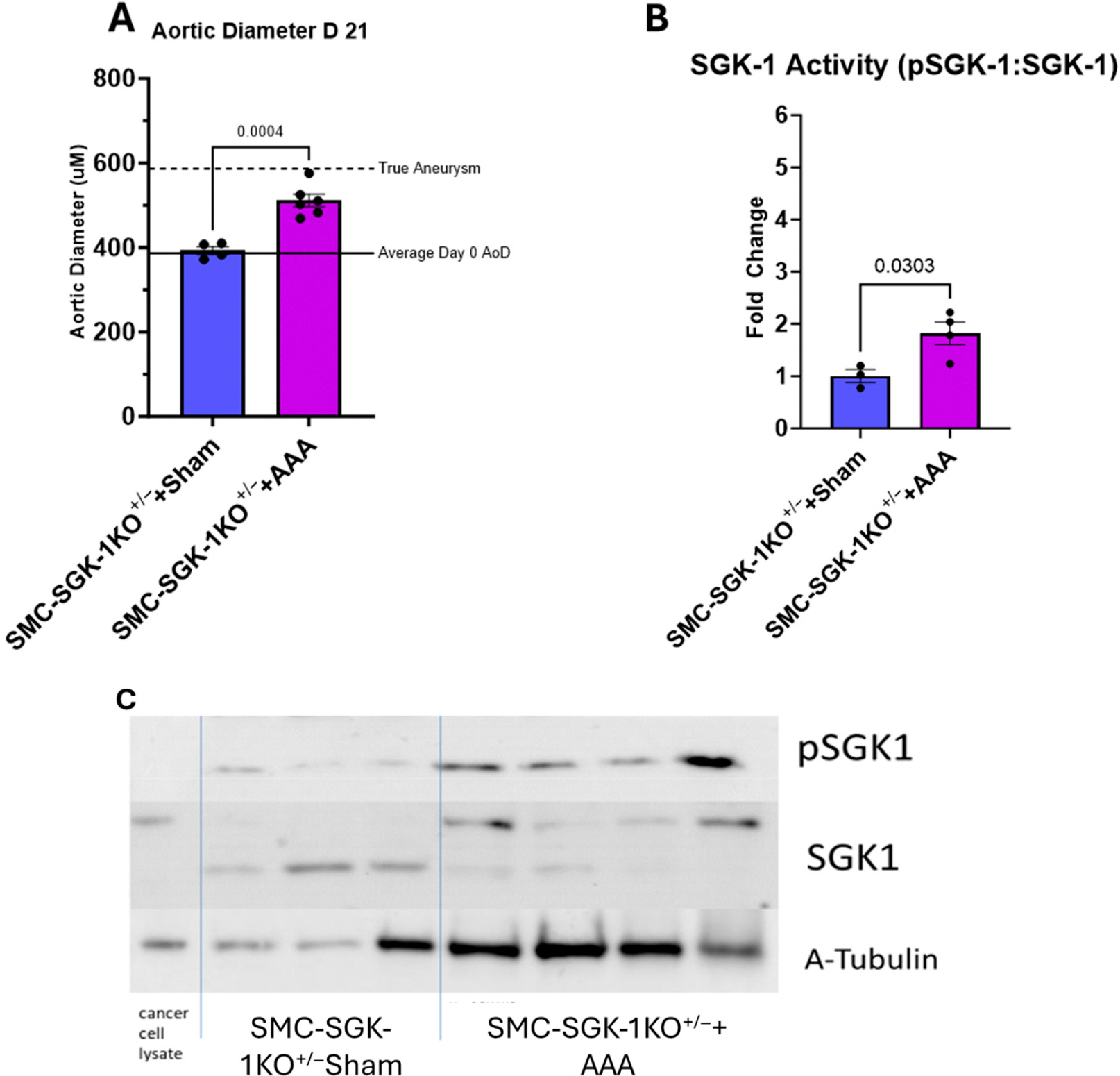
Panel (**A**): Aortic diameter (AoD) of SMC-SGK-1KO+/− mice +/− AAA induction at day 21. Solid black line represents average baseline aortic diameter on day 0. Dotted black line represents an average 50% increase in diameter to represent the threshold of a true AAA. Panel (**B**): SGK-1 activity represented as fold change from C57Bl/6+Sham. Panel (**C**): Representative Western blot indicating abundance of pSGK-1 versus SGK-1 and alpha-tubulin. Statistical analysis represents Student’s *t*-test.

To interrogate whether our individual transgenic strains carried aortic physiology in line with the C57Bl/6 background in preparation for experimentation with the SMC-SGK1-KO^+/−^ mice, Sham versus AAA induction was conducted in MyH11^Cre-EGFP^ (Supplemental Figure S1A) and SGK-1^flox/flox^ (Supplemental Figure S1B) mice with terminal assessment at 21 days. Selected mice were also treated with EMD infusion and trends in AoD followed that of C57Bl/6 mice such that AAA development was attenuated with EMD therapy, concurrently providing additional evidence that SGK-1 drives AAA growth and supporting the relevance of the SMC-SGK1-KO^+/−^ strain in this experimentation. AoD increased to 510.99 μM ± 15.25 μM in SMC-SGK1-KO^+/−^+AAA mice, representing an average diameter growth of 28.20% ± 3.74% as compared to the SMC-SGK1-KO^+/−^+Sham mice (Figure 2A). While this diameter expansion did represent a significant increase from baseline (*p* = 0.004), true AAA was not established. Importantly, the SMC-SGK1-KO^+/−^+AAA mice did demonstrate increased SGK-1 activity compared to SMC-SGK1-KO^+/−^+Sham (Figure 2B,C; 1.82 ± 0.43-fold; *p* = 0.0303), and that value was comparable to SGK-1 activity noted in the C57Bl/6+AAA+EMD group. These findings suggest that SGK-1 activity was upregulated through this method of AAA induction, particularly in VSMCs, but a minimum threshold of SGK-1 activity may be needed to develop true AAA.

### 3.2. Aortic Biomechanical Changes Following AAA Induction

**Figure 3.**
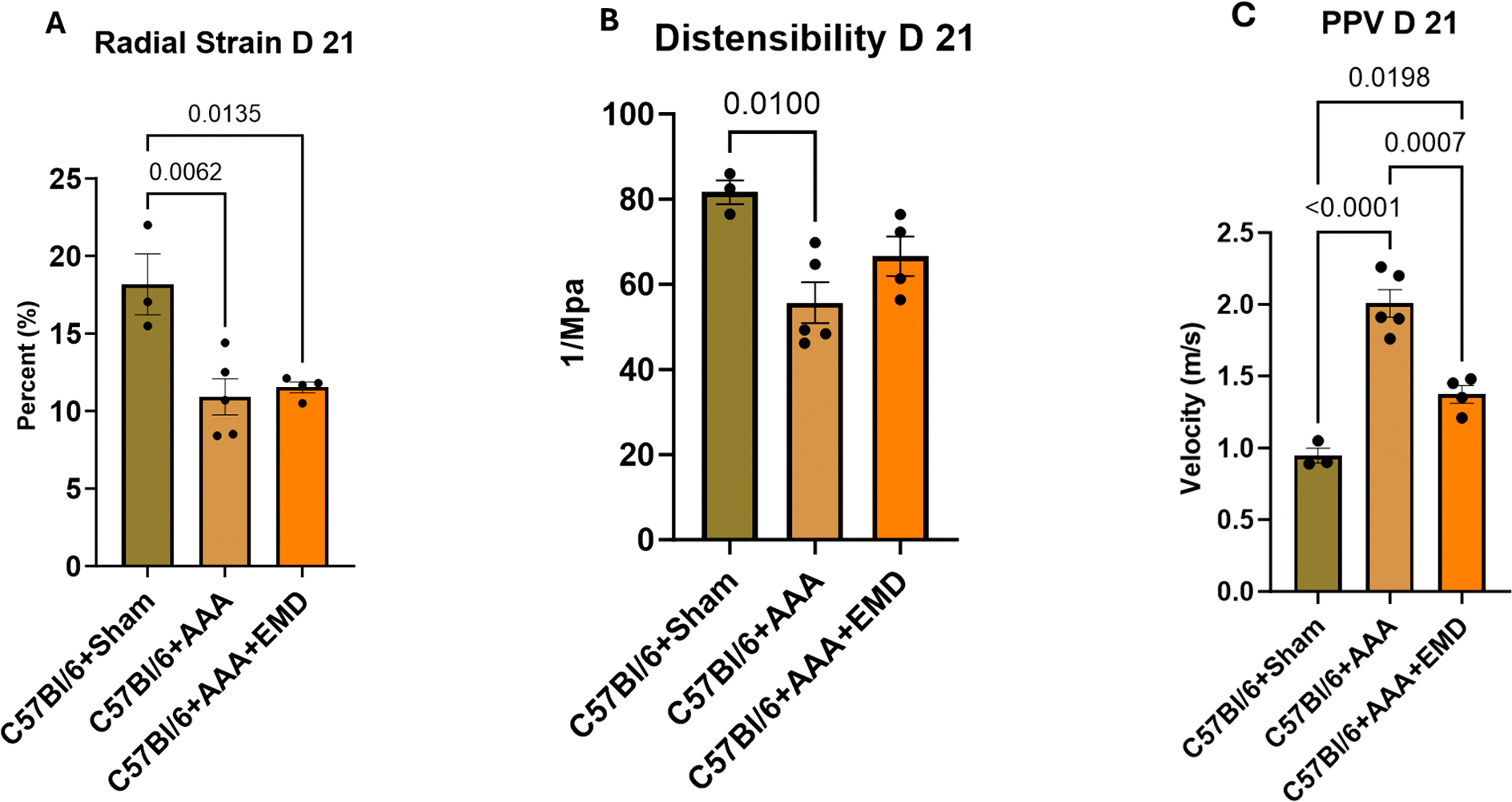
Ultrasound-derived mechanical parameters for C57Bl/6 mice +/− AAA induction and +/− EMD therapy at day 21 represented as Panel (**A**): radial strain, Panel (**B**): distensibility, and Panel (**C**): pulse propagation velocity (PPV). Statistical analysis represents ANOVA.

Radial strain was significantly reduced in the C57Bl/6+AAA (*p* = 0.0062) and C57Bl/6+AAA+EMD (*p* = 0.0135) groups when compared to their C57Bl/6+Sham counterparts (Figure 3A), indicating increased aortic stiffness. Distensibility showed a similar pattern of change such that there was a significant decrease among C57Bl/6+AAA mice (Figure 3B; *p* = 0.01 vs. C57Bl/6+Sham), which again represents an increase in aortic stiffness and these changes are likely related to the known alteration in matrix composition during AAA development. Regarding PPV, this assessment of vascular mechanics was significantly elevated in the C57Bl/6+AAA mice (*p* < 0.0001 vs. C57Bl/6+Sham) but inhibited by EMD therapy (*p* = 0.0007 vs. C57Bl/6+AAA), suggesting that SGK-1 activity specifically contributes to the loss of functional vascular resistance.

**Figure 4.**
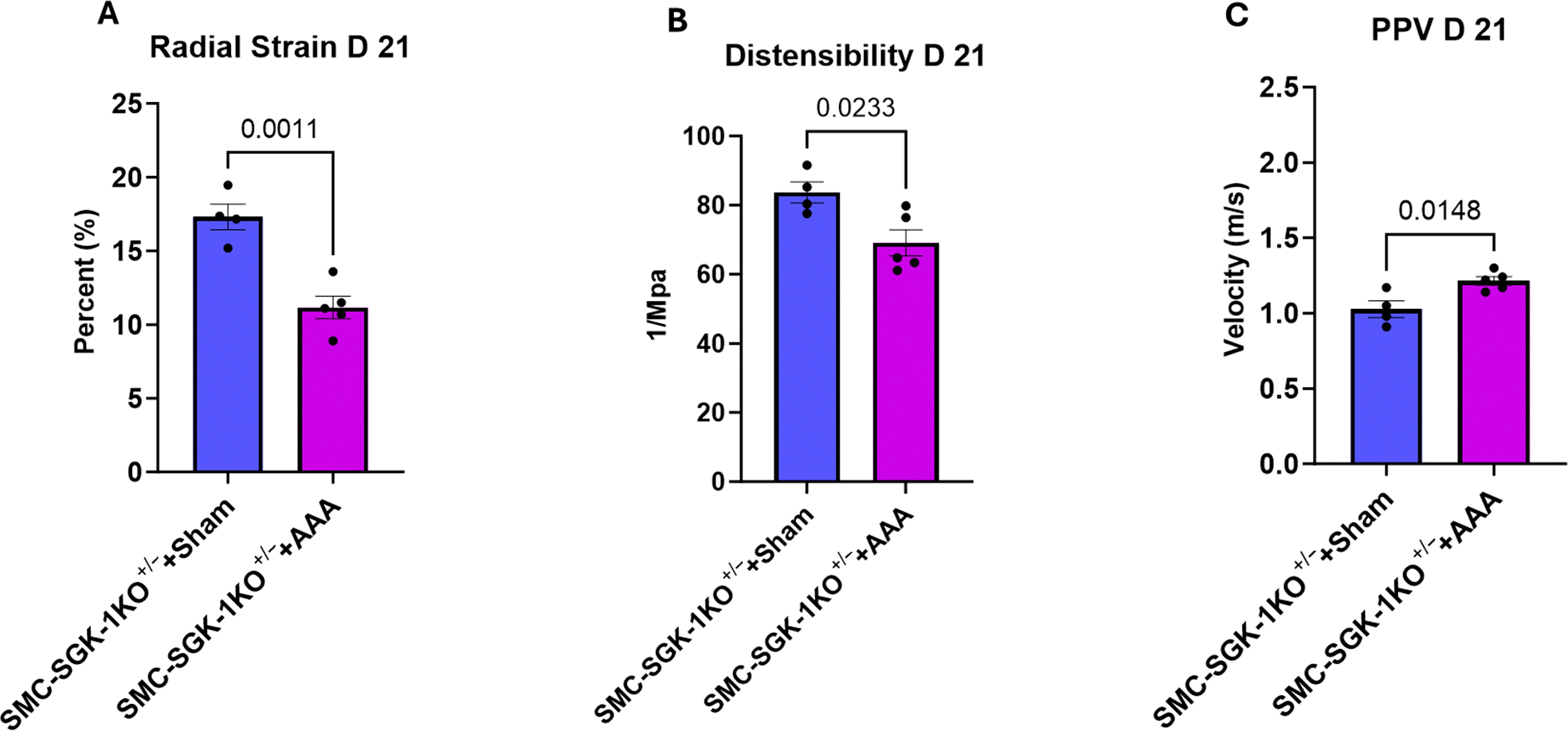
Ultrasound-derived mechanical parameters for SMC-SGK-1KO^+/−^ mice +/− AAA induction at day 21 represented as Panel (**A**): radial strain, Panel (**B**): distensibility, and Panel (**C**): PPV. Statistical analysis represents Student’s *t*-test.

Again, to confirm the validity of the SMC-SGK1-KO^+/−^ strain, aortic biomechanical parameters were assessed in the progenitor strains with AAA induction. MyH11^Cre-EGFP^ (Supplemental Figure S2) and SGK-1^flox/flox^ (Supplemental Figure S3) mice demonstrated significant aortic stiffness in AAA as quantified by radial strain, distensibility, and PPV. SMC-SGK1-KO^+/−^+AAA mice showed reductions in radial strain (Figure 4A; *p* = 0.0011 vs. SMC-SGK1-KO^+/−^+Sham) and distensibility (Figure 4B; *p* = 0.0233 vs. SMC-SGK1-KO^+/−^+Sham) representative of matrix alterations driving aortic stiffness. Although statistically elevated from the SMC-SGK1-KO^+/−^+Sham scenario, PPV among the SMC-SGK1-KO^+/−^+AAA showed a modest interval increase which was similar to the absolute value captured in the C57Bl/6+AAA+EMD group above, thereby supporting SGK-1 as a target to maintain a component of aortic function. More specifically, by recapitulating these findings in the smooth muscle cell-specific knockout mice, it appears that SGK-1 activity is impacting aortic VSMC function during AAA growth.

## 4. Discussion

SGK-1 activity has been documented in high-pressure remodeling of several vascular beds, and our group has explored its role in hypertensive inflammatory infiltration as a contributor to atherosclerotic plaque deposition, but this investigation approached the unique question of how SGK-1 activity impacted AAA growth and correlated to stiffness. Using a previously validated periadventitial CaCl_2_ model of murine AAAs with ultrasound-derived assessment of aortic stiffness parameters, we have distinctly demonstrated that AAA growth can be attenuated with either pharmacologic or genetic inhibition of SGK-1. While matrix-associated aspects of stiffness were unaffected, reduction in SGK-1 activity coincided with recovery of PPV toward baseline which suggests regained aortic VSMC function. These findings highlight the role of SGK-1 in AAA formation and aortic stiffness, thereby supporting ongoing investigation into the potential benefit of SGK-1 inhibition in patients with AAA.

SGK-1 expression is highly variable and can be subject to a wide range of triggers and hormonal mediators [21]. The described mechanosensitivity of SGK-1 has led investigators, such as ourselves, to initially pursue experimentations related to hypertension, thereby demonstrating elevated SGK-1 activity in mechanical and hormonal rodent models of elevated blood pressure [11,12,22]. More specifically, SGK-1 was recognized as a critical component of the mechanotransduction signaling cascades within the vascular wall to influence matrix remodeling [11,23]. Extending that concept to the substantial therapeutic gap for medical management of AAA, we then chose to apply this tension-induced signaling concept to explore drivers of aortic degenerative remodeling. Population-based studies have shown that aortic stiffness is significantly higher in patients with AAA [24], and changes in the extracellular matrix are described as the driving force behind both increased aortic stiffness and AAA development [25–27]. However, we propose that the stiffness is not just a result of the AAA, but that matrix forces also serve as mechanical stress on the aortic medial VSMC to affect their synthetic function and promote AAA growth. Within this model, the reduction in both radial strain and distensibility following AAA induction are evidence of matrix-related stiffness. When uninterrupted, the model proceeds to generate small AAA, but both pharmacologic and genetic knockdown of SGK-1 reduced that dilation to only about ~25% beyond baseline. Attributing that initial growth and stiffness to the early inflammatory infiltrate and protease production following peri-adventitial CaCl_2_ application [28], these findings suggest that SGK-1-dependent signaling in the medial VSMCs was upregulated due to the intrinsic matrix forces and therefore inhibition was able to quench AAA development.

As an alternative to radial strain and distensibility which rely heavily on matrix integrity, pulse propagation velocity (PPV) represents a measurement of the speed at which a pulse travels down the aortic wall and relies heavily on VSMC-derived vascular tone [29]. Previous research using both murine AAA models as well as patient imaging has shown elevated PPV in the presence of AAA, a functional sign of aortic stiffness [30–32]. In this experimentation, again PPV was elevated in AAA, but preserved with SGK-1 inhibition, expecting to represent maintenance of the healthy contractile VSMC phenotype. Within the known association of VSMC phenotype switching and AAA development, the reduced contractile force of synthetic VSMCs results in aortic diameter increase while the disruption to the elastic lamellae and dysfunctional connection between the VSMCs and extracellular matrix results in altered aortic compliance [33]. A similar phenomenon has recently been described in the pathophysiology of thoracic aortic dissection [34]. Characterization of the VSMC phenotypes was not pursued in this experimentation, but utilization of ultrasound to assess PPV specifically within the infrarenal region provides strong evidence that inhibition of SGK-1 impacted function of the local medial VSMCs to stifle aortic dilation and prevent development of AAA. Considering the widespread utilization of ultrasound to assess small AAA, these findings carry significant clinical relevance and warrant further investigation.

### Limitations

While this experimentation carries notable clinically relevant strengths such as (1) uniquely indicating that elevated activity of SGK-1 in VSMCs can promote AAA growth, and (2) inhibiting SGK-1 through pharmacologic or genetic knockdown reduced aortic dilation along with improved contractile function of VSMC as evidenced by reduced PPV, there are limitations to be addressed. With a focus on evaluating how SGK-1 influenced AAA growth, this project utilized a single timepoint with introduction of the selective inhibitor EMD638683 at the time of AAA induction. While this model was a reasonable correlate to the constitutive reduction in SGK-1 activity in SMC-SGK1-KO^+/−^ mice, it does not account for the clinically relevant aspect of initiating therapy after a small AAA has formed. These time-dependent aspects of SGK-1 activity will be addressed in future investigations. Similarly, off-target effects of EMD therapy were not grossly observed over this relatively short treatment time in the mice, but impact on non-vascular system will be integrated. Having expected to derive SGK-1 impact on inflammatory matrix remodeling but instead identified potential for VSMC functional maintenance, this experimentation did not include direct assessment of VSMC phenotype markers, but this interesting aspect will be further explored. Due to the lethality of homozygous knockout in the SMC-SGK-1KO strain, dual inhibition of SGK-1 by pharmacologic and genetic means was not pursued with expected high animal mortality. Combined therapeutic approaches will be addressed following the construction of an inducible SMC-SGK-1KO strain. As these details are integrated into ongoing investigations, we look forward to fully defining the translational opportunity to inhibit SGK-1 to reduce AAA growth.

## 5. Conclusions

In this model of murine AAA, SGK-1 inhibition attenuated AAA growth and preserved vascular function. Pharmacologically targeting this pathway may be an exciting directed medical therapy for AAA and requires further investigation.

## Supplementary Material

Supplement

**Supplementary Materials:** The following supporting information can be downloaded at: https://www.mdpi.com/article/10.3390/jvd5010010/s1, Figure S1: Aortic diameter (AoD) +/− AAA induction and +/− EMD treatment at day 21 of (A) MyH11^Cre-EGFP^ and (B) SGK-1^flox/flox^ mice; Figure S2: Ultrasound-derived mechanical parameters of MyH11^Cre-EGFP^ mice +/− AAA induction at day 0 versus day 21; Figure S3: Ultrasound-derived mechanical parameters of SGK-1^flox/flox^ mice +/− AAA induction at day 0 versus day 21.

## Data Availability

The raw data supporting the conclusions of this article will be made available by the authors on request.

## Abbreviations

The following abbreviations are used in this manuscript:

AAA: Abdominal Aortic Aneurysm
VSMC: Vascular Smooth Muscle Cell
HTN: Hypertension
SGK-1: Serum and Glucocorticoid Inducible Kinase-1
SMC-SGK1-KO^+/−^: Heterozygous Smooth Muscle Cell-Specific SGK-1 Knockout Strain
AoD: Aortic Diameter
CaCl_2_: Calcium Chloride
PPV: Pulse Propagation Velocity
pSGK-1: Phosphorylated SGK-1
SEM: Standard Error of Mean
