# Supplementary material for: Smooth Muscle Cell Specific Activity of SGK-1 Alters Aortic Stiffness and Abdominal Aortic Aneurysm Growth
Source: J Vasc Dis. Author manuscript; Available in PMC 2026 Apr 21. (PMC13095228; doi:10.3390/jvd5010010)
Supplement: Supplement [file NIHMS2156759-supplement-Supplement.pptx]

## Slide 1
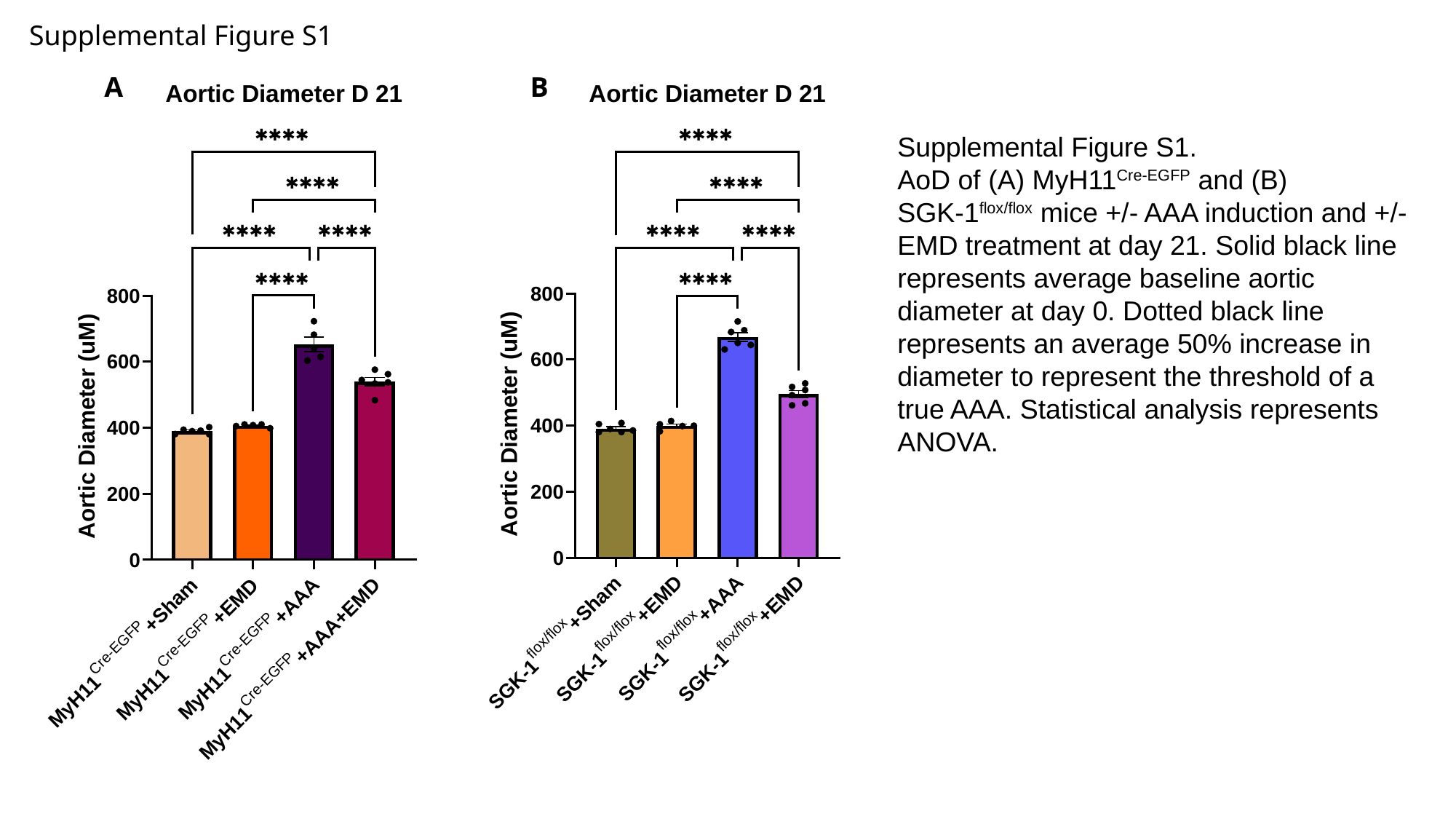

Supplemental Figure S1
A
B
Supplemental Figure S1.
AoD of (A) MyH11Cre-EGFP and (B) SGK-1flox/flox mice +/- AAA induction and +/- EMD treatment at day 21. Solid black line represents average baseline aortic diameter at day 0. Dotted black line represents an average 50% increase in diameter to represent the threshold of a true AAA. Statistical analysis represents ANOVA.

## Slide 2
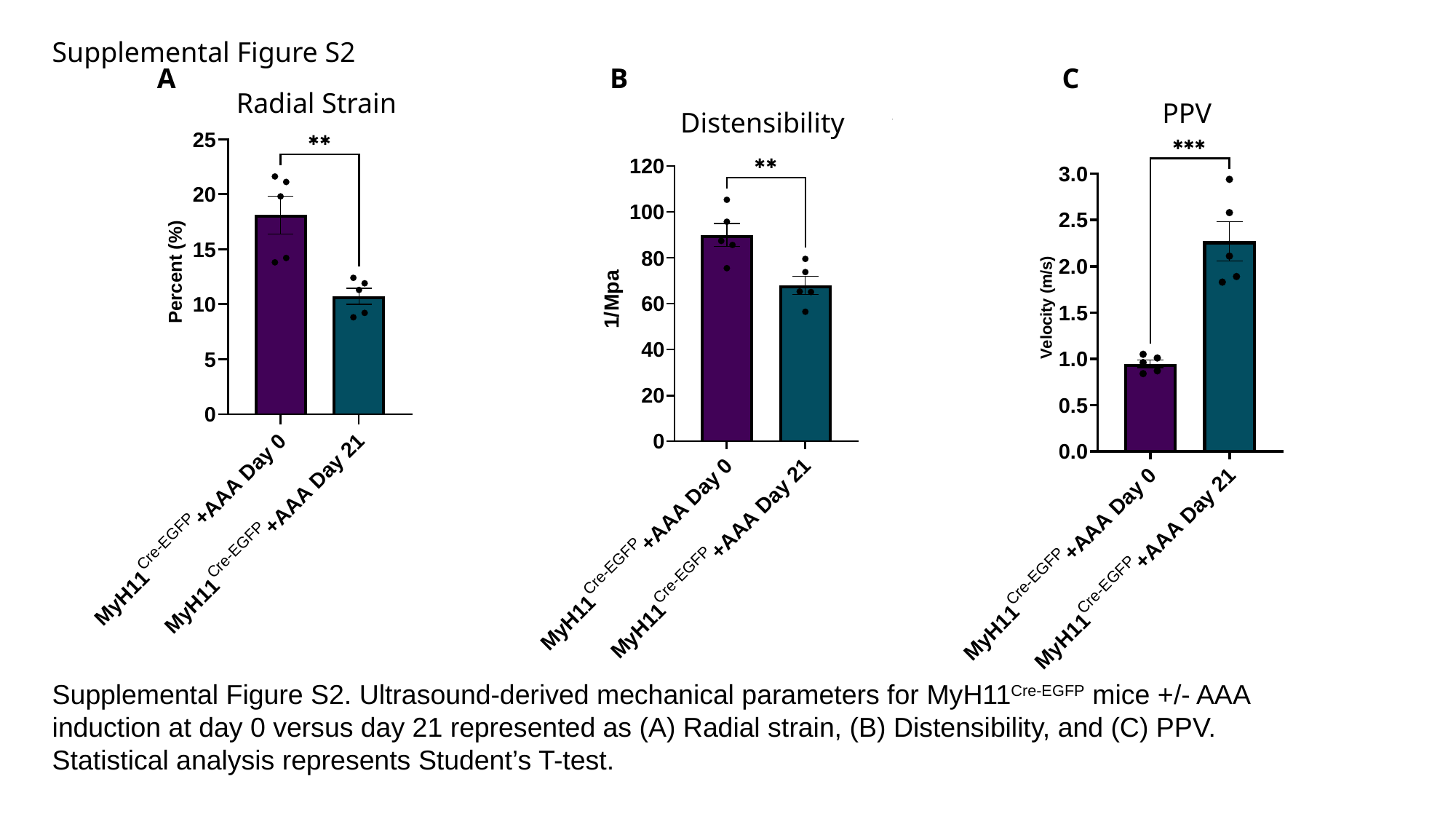

Supplemental Figure S2
B
C
A
Radial Strain
PPV
Distensibility
Supplemental Figure S2. Ultrasound-derived mechanical parameters for MyH11Cre-EGFP mice +/- AAA induction at day 0 versus day 21 represented as (A) Radial strain, (B) Distensibility, and (C) PPV. Statistical analysis represents Student’s T-test.

## Slide 3
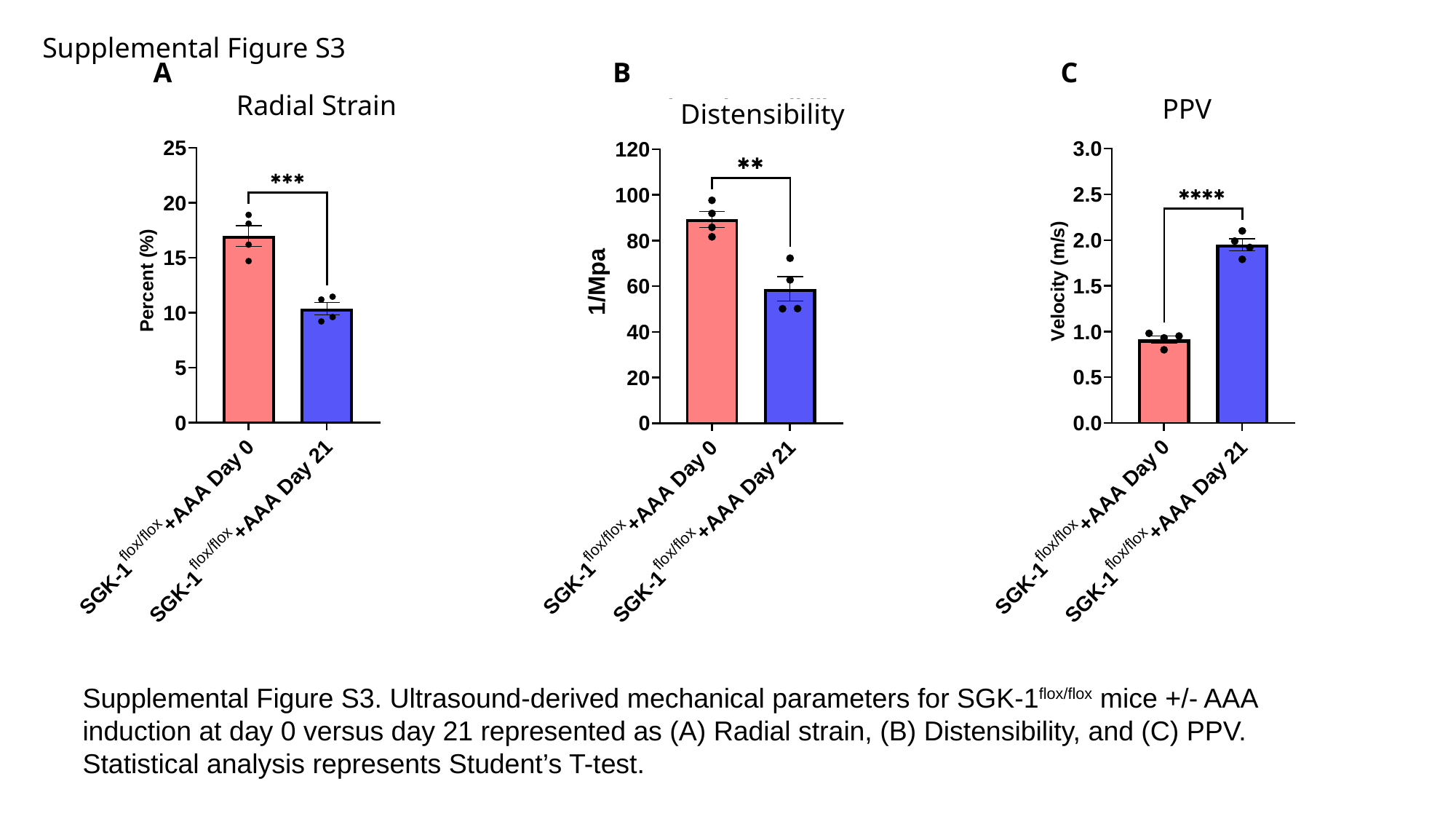

Supplemental Figure S3
A
B
C
Radial Strain
PPV
Distensibility
Supplemental Figure S3. Ultrasound-derived mechanical parameters for SGK-1flox/flox mice +/- AAA induction at day 0 versus day 21 represented as (A) Radial strain, (B) Distensibility, and (C) PPV. Statistical analysis represents Student’s T-test.
